# Human Adult Stem Cells Maintain a Constant Phenotype Profile Irrespective of Their Origin, Basal Media, and Long Term Cultures

**DOI:** 10.1155/2015/146051

**Published:** 2015-01-20

**Authors:** Indumathi Somasundaram, Rashmi Mishra, Harikrishnan Radhakrishnan, Rajkumar Sankaran, Venkata Naga Srikanth Garikipati, Dhanasekaran Marappagounder

**Affiliations:** ^1^Department of Stem Cells, National Institute of Nutrition, Secunderabad 500 007, India; ^2^Institute for Biochemistry and Molecular Biology, Ulm University, M24, Level 3, Room 358, Albert-Einstein-Allee 11, 89081 Ulm, Germany; ^3^Berlin School of Integrative Oncology, Buch, 131254 Berlin, Germany; ^4^Lifeline RIGID Hospitals, Chennai 600 010, India; ^5^Stem Cell Therapy Program, Center for Translational Medicine, Temple University School of Medicine, Temple University, Room MERB 9-943, 3500 North Broad Street, Philadelphia, PA 19104, USA; ^6^Ree Laboratories Private Limited, Andheri West, Mumbai 400 053, India

## Abstract

The study aims to identify the phenotypic marker expressions of different human adult stem cells derived from, namely, bone marrow, subcutaneous fat, and omentum fat, cultured in different media, namely, DMEM-Low Glucose, Alpha-MEM, DMEM-F12 and DMEM-KO and under long term culture conditions (>P20). We characterized immunophenotype by using various hematopoietic, mesenchymal, endothelial markers, and cell adhesion molecules in the long term cultures (Passages-P1, P3, P5, P9, P12, P15, and P20.) Interestingly, data revealed similar marker expression profiles irrespective of source, basal media, and extensive culturing. This demonstrates that all adult stem cell sources mentioned in this study share similar phenotypic marker and all media seem appropriate for culturing these sources. However, a disparity was observed in the markers such as CD49d, CD54, CD117, CD29, and CD106, thereby warranting further research on these markers. Besides the aforesaid objective, it is understood from the study that immunophenotyping acts as a valuable tool to identify inherent property of each cell, thereby leading to a valuable cell based therapy.

## 1. Introduction

The ubiquitous existence of multipotent mesenchymal stem cells annexes to be a powerful regenerative tool for its use in cellular therapeutics rendering the replacement of worn out cells [1, 2]. Despite the recent advancement, stem cell therapy is still at its infancy, attributed with several hurdles in regenerative applicability. This might be due to the lack of an ideal source of stem cells that accounts for the functional improvement of the diseased. The isolation and applicability of stem cells derived from the prehistoric source, human bone marrow, and the contemporary source of human adipose tissue has revolutionized the field of regenerative medicine [3–5]. Although these sources outweigh certain uncertainties, stem cell therapeutics in many cases was unsuccessful [6, 7]. The rationale of this failure in terms of stem cell survival, proliferation, and regeneration remains unclear.

Although the reason for the same is not fully understood, researchers combat towards overcoming the recognized barriers such as hyperglycemia, hypoxia and inflammation to maximize the beneficial effects of MSC in cellular therapeutics [8, 9]. However, yet another potential reason for such failure might be due to the lack of understanding the individual components innate capability that forms the basis of tissue maintenance, repair, and regeneration. This is attributed to the fact that stem cells of adipose tissue and bone marrow reside in a more heterogeneous crude mixture along with the other constituents such as loose connective tissue matrix, endothelial cells, vascular smooth muscle cells, pericytes, leucocytes, mast cells, mesenchymal stem cells, and immune cells such as resident hematopoietic progenitor cells and macrophages [10–12]. The in vitro characterization and maintenance of these heterogenous tissue stem/progenitor cells are critical aspects when assessing their potential for clinical application. It is a well-known fact that stem cells use their receptors for binding other signalling molecules as a way of communication to carry out their functions of self-renewal and differentiation. Despite several attempts of research efforts on revealing their biological properties [10, 13], the phenotypic and functional characteristics of these stem cells, to date, still remain obscure.

The rationale behind this ambiguity relies on the hypothesis that influence of different media and media composition may lead to variations in marker expression [14]. In addition, it is also reported that these markers may or may not be evident at primitive stages or may get lost with expansion in vitro or in vivo [15], thereby identity of inherent population for therapeutic interventions becomes a strenuous task. These discrepancies based on phenotypic characterization of MSCs make its applicability indefinite, thereby demanding a quest for identification of prospective definitive marker profiles of MSCs in vitro. Being in the regenerative medicine epoch of treatment of degenerative diseases, it is important to address this inconclusive tribulation. Hence, identification of prospective markers of most widely used sources such as adipose tissue and bone marrow is of utmost importance to address the following reasons. Firstly, to understand the innate capability of each cell population according to its surface expression pattern, secondly, to advance our understanding of basic biological processes of stem cells during self-renewal and differentiation, that is, their in vivo functionality and finally, to demarcate and develop valuable cell based therapies.

In lieu of the above, this study aimed to identify whether the phenotypic marker expression profiles vary between sources such as bone marrow and subcutaneous fat under different media (DMEM-Low Glucose, Alpha-MEM, DMEM-F12, and DMEM-KO) and under long term culture conditions (>P20). Omentum fat is also included in the study as its immense potency is also underway [16–19].

## 2. Materials and Methods

### 2.1. Sampling

The protocol followed for all samples was reviewed and approved by the hospital review board and ethics committee of Lifeline Multispecialty Hospital, Chennai, India. The samples were collected in-house and the research pursuit was explained to the patients followed by obtaining a written informed consent prior to collection of samples.

The omentum fat was collected from patients undergoing exploratory laparotomy. The omentum fat biopsies of 25–40 g were obtained from 4 subjects (*n* = 4) with age group ranging from 28 to 50 and mean BMI of 26.5 ± 2.1 kg/m^2^. The collected tissues were processed within 4 hours of removal of fat from patients.

The subcutaneous fat was collected from obese patients undergoing Bariatric surgery in the form of abdominoplasty. Subcutaneous fat of 25–50 g was obtained from 4 subjects (*n* = 4) with age group ranging from 35–55 with a BMI 27.3 ± 1.8 kg/m^2^ after completion of surgery. The tissues were quantified and were processed within 4 hours of collection.

Human bone marrow samples were obtained from the iliac crest of patients (*n* = 4) undergoing experimental stem cell therapy for spinal cord injury with mean age of 35.3 ± 3.33 and body mass index (BMI) of 23.5 ± 1.167. All the samples were processed within 2 hours of collection.

### 2.2. Cell Isolation 

#### 2.2.1. Bone Marrow

Mononuclear cells were isolated from bone marrow aspirate by density gradient centrifugation using Ficoll Paque. The aspirates were diluted with twice the amount of PBS (Invitrogen) and layered on to Ficoll Paque (Stemcell Technologies) solution in a centrifuge tube. The layered samples were further centrifuged and the buffy coat layer containing the mononuclear cells was collected. The isolated mononuclear cell suspension was washed with PBS to remove the residual Ficoll content and other contaminants. The erythrocyte content in the isolated pellet was lysed using 0.7% NH_4_Cl and lysis reaction was stopped with 0.9% ice cold NaCl. The suspension was centrifuged to obtain the mononuclear cell fraction. The isolated cells were further resuspended in PBS and its total cell count and viability were determined by trypan blue exclusion method.

#### 2.2.2. Adipose Tissue

The surgical samples of obtained subcutaneous fat and omentum fat were washed thrice in wash buffer 1x Phosphate Buffered Saline (PBS) (Hi-Media) containing 1% antibiotic-antimycotic solution (Invitrogen) and were minced into 2-3 mm in diameter. These minced pieces were further digested by 0.075% collagenase type-1 (Hi-Media) solution. 10% Fetal Bovine Serum (FBS) (Invitrogen) was used to inhibit the activity of collagenase. The digested cells were centrifuged at 600 g for 10 minutes at 20°C. The stromal vascular fraction found in the pellet obtained was washed further and subjected to erythrocyte lysis using 0.7% NH_4_Cl solution for 5 minutes at room temperature. The cells were subjected to further centrifugation and the pellet recovered was resuspended in PBS. A single cell suspension was obtained after filtration after which the cell viability was evaluated using Trypan Blue staining.

### 2.3. Cell Culture

Cells isolated from these aforesaid sources were plated at a density of 3 × 10^5^/25 cm^2^ flask (Nunc) and cultured in four different filter sterilized media: DMEM-LG (Invitrogen), *α*-MEM (Invitrogen), DMEM-F12 (Invitrogen), and DMEM-KO (Invitrogen), each of which was supplemented with 10% FBS (Invitrogen) and 1% antibiotic-antimycotic solution. The cells were maintained for 2–4 days before first media change. Standard culture conditions of 37°C, 5% CO_2_, and 95% humidity were maintained and 70–80% confluency was obtained. The primary culture was subcultured until passage 20 with media changes twice every week.

### 2.4. FACS Analysis

Flowcytometric characterization was performed using Becton, Dickinson FACS Aria (BD FACS Aria). Approximately 1 × 10^6^ cells were stained with saturating concentrations of fluorochrome conjugated antibodies, CD34 PE (BD Biosciences), CD45 APC CY7 (BD Biosciences), CD133 APC (e-Biosciences), CD31 FITC (BD Biosciences), HLADR PERCP (BD Biosciences), CD44 FITC (BD Biosciences), CD73 PE (BD Biosciences), CD13 APC (BD Biosciences), CD29 PE (BD Biosciences), CD90 PERCP (e-Biosciences), CD105 APC (e-Biosciences), SSEA4 ALEXAFLOUR (e-Biosciences), CD117 APC (e-Biosciences), ABCG2 PE (e-Biosciences), CD166 PE (BD Biosciences), CD106 FITC (BD Biosciences), CD54 PERCP (BD Biosciences), CD 49d PE (e-Biosciences), and ALDH. The cells were incubated in the dark for 20 minutes at 37°C. The incubated cells were washed thrice with wash flow buffer consisting of phosphate buffer supplemented with 2% (v/v), FBS (Sigma Aldrich), and 0.1% (w/v) sodium azide, NaN_3_ (Sigma Aldrich) and resuspended in 500 *μ*L of BD FACS flow and vortexed. BD FACS-DIVA Software was used for sample data acquisition and analysis. The first plot is created with FSC versus SSC in all experiments. The subsequent plots were created using the respective flourochrome (*x* axis) along with SSC (*y* axis). The FSC Vs SSC were created to identify the different cell population and to avoid debris. The isotype control was used to set the gates and the analysis regions. The readings of each antibody cocktail in respective tubes were run, analysed, and recorded. All samples were characterized and recorded with a minimum of 10000 events.

### 2.5. ALDH Analysis

ALDH analysis was performed using Aldehyde dehydrogenase kit (Stem Cell Technologies). Dry ALDEFLUOR reagent was activated by incubating with DMSO followed by incubation with 2 N HCl at room temperature. The incubated mixture was further added with ALDEFLUOR assay buffer and stored at −20°C. Briefly, 1 × 10^5^ cells were recovered by centrifugation and resuspended in assay buffer (1 × 10^5^ cells per mL). The suspended sample was treated with 5 *μ*L of activated ALDH substrate at 37°C in water bath. The incubated sample was further centrifuged and the pellet obtained was resuspended in cold ALDEFLUOR assay buffer. The stained cells were analysed in a flowcytometer with FITC channel. A sample tube containing DEAB (Diethylaminobenzaldehyde—a specific inhibitor of ALDH) was ran as control.

### 2.6. Statistical Analysis

All data obtained from omentum fat, subcutaneous fat, and bone marrow samples in different media (*n* = 4) were represented as Mean ± Standard Error Mean (SEM). The data were analysed using One Way Analysis of Variance (ANOVA) along with Duncan multiple range test using SPSS 15.0 (SPSS Inc., Chicago, IL, USA). *P* values were calculated to determine the statistically significant variations. Results were considered statistically significant when *P* < 0.05 and *P* < 0.01.

## 3. Results

### 3.1. Comparative Expression Profiles of Collated Surface Antigens

**Figure 1:**
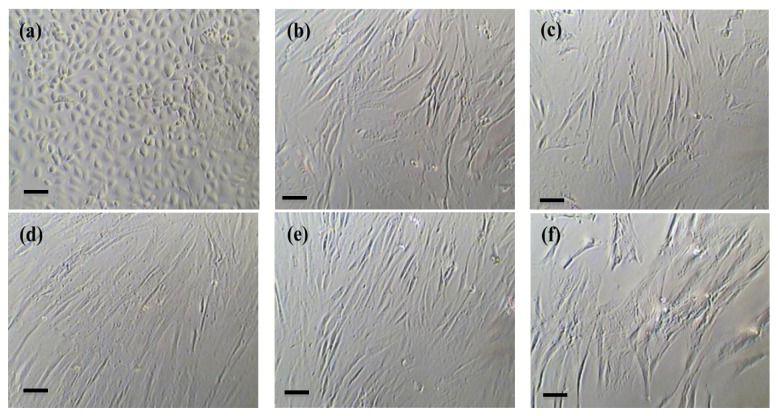
Morphology of cultured mesenchymal stem cells. Representative photomicrographs showing morphology of mesenchymal stem cells at early passage (P3) derived from omentum fat (a), subcutaneous fat (b), and bone marrow (c) and at late passage (P20) derived from omentum fat (d); subcutaneous fat (e) and bone marrow (f), (Scale-20 *μ*m; Magnification at 20x).

The MSCs of early and later passages from omentum fat, subcutaneous fat, and bone marrow cultured extensively (until P20) in four different media as illustrated (Figure 1) were phenotypically characterized (*n* = 4) for the diverse panel of cell surface marker profiles including mesenchymal stem cell, CD90, CD73, and CD105 as proposed by the International Society for Cell Therapy (ISCT), hematopoietic stem cell, CD34, CD45, and CD133, cell adhesion molecules, CD29, CD49d, CD44, CD166, CD106, CD54, and CD31, and certain unique markers such as CD13, CD117, HLADR, ABCG2, CD140b, SSEA4, and ALDH using flowcytometry.

**Figure 2:**
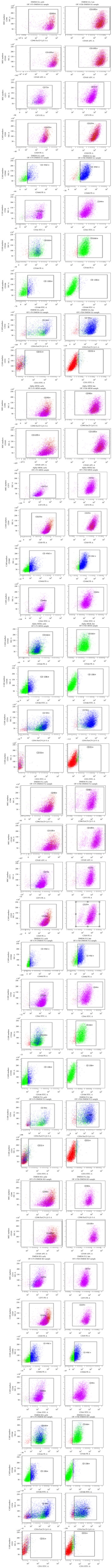
Flowcytometric characterization of MSCs and CAM of omentum fat derived MSC. Characterization of omentum fat derived MSC at early (P3) and late (P20) passages in DMEM-LG, Alpha-MEM, DMEM-F12, and DMEM-KO for CD90, CD105, CD73, CD29, CD49d, CD44, CD166, CD106, CD54, and CD31 using flowcytometry.

**Figure 3:**
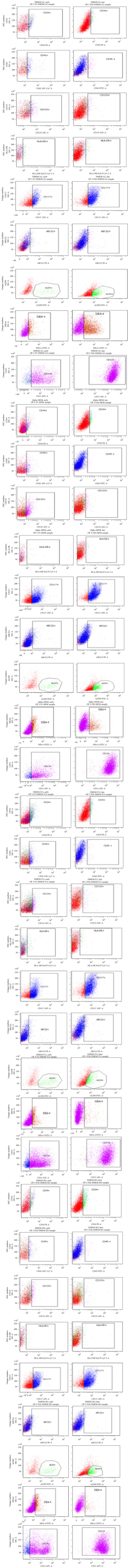
Flowcytometric characterization of HSC and unique markers of omentum fat derived MSC. Characterization of omentum fat derived MSC at early (P3) and late (P20) passages in DMEM-LG, Alpha-MEM, DMEM-F12, and DMEM-KO for CD34, CD45, CD133, HLA-DR, CD117, ABCG2, ALDH, SSEA4, and CD13 using flowcytometry.

**Figure 4:**
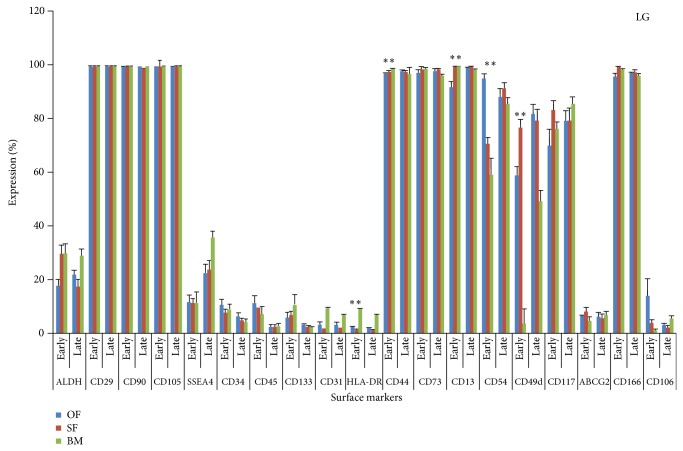
Marker expression profiling of MSCs cultured in DMEM-Low Glucose (DMEM-LG). Comparative Surface antigenic profiling of MSCs derived from omentum fat, subcutaneous fat, and bone marrow at early (P3) and late (P20) passages cultured in DMEM-LG.

**Figure 5:**
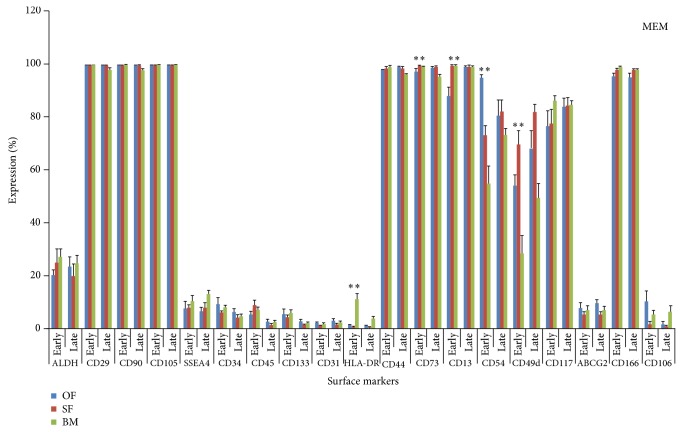
Marker expression profiling of MSCs cultured in Alpha-MEM. Comparative Surface antigenic profiling of MSCs derived from omentum fat, subcutaneous fat, and bone marrow at early (P3) and late (P20) passages cultured in Alpha-MEM.

**Figure 6:**
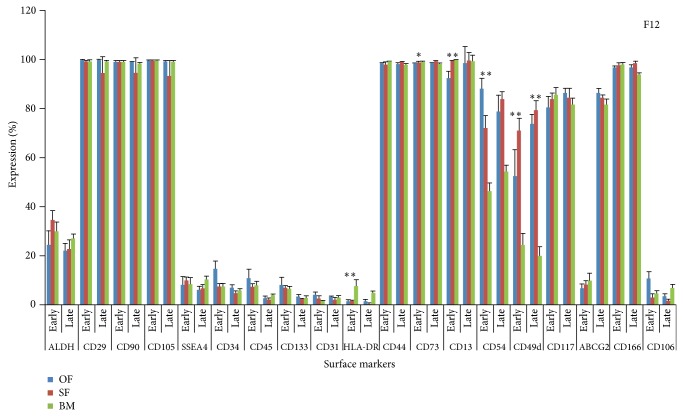
Marker expression profiling of MSCs cultured in DMEM-F12. Comparative Surface antigenic profiling of MSCs derived from omentum fat, subcutaneous fat, and bone marrow at early (P3) and late (P20) passages cultured in DMEM-F12.

**Figure 7:**
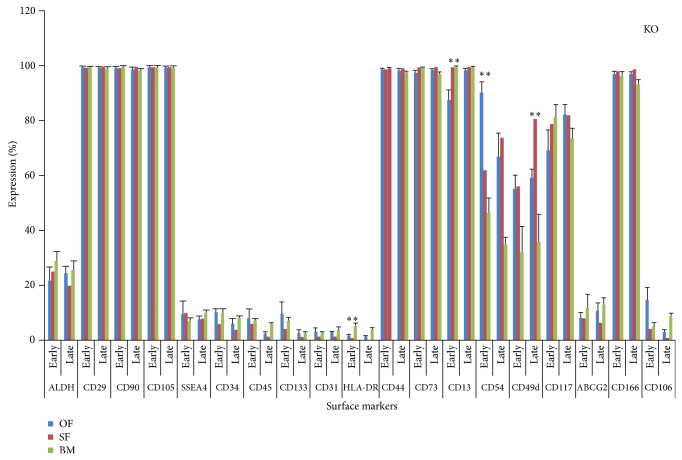
Marker expression profiling of MSCs cultured in DMEM-KO. Comparative Surface antigenic profiling of MSCs derived from omentum fat, subcutaneous fat, and bone marrow at early (P3) and late (P20) passages cultured in DMEM-KO.

The dotplots of flowcytometric analysis of these diversified markers for omentum fat (Figures 2 and 3) were illustrated. The comparison of surface antigenic expression profiles of cultured MSCs at early and later passages of these aforesaid sources in DMEM-LG (Figure 4), Alpha-MEM (Figure 5), DMEM-F12 (Figure 6), and DMEM-KO (Figure 7) were comprehended graphically in the form of Mean ± SEM with its statistical significance. Despite the similarities in most of the markers in all media, some inconsistent expressions were identified in markers such as CD117, CD54, and CD49d.

For ease of comparative expression analysis, the articulation of the marker expression was made according to previously specified range and category (Table 1) such as: Remarkable (90–100%), high expression (75–89%), moderate expression (40–74%), low expression (11–39%) and sparse expression (1–10%) [20].

### 3.2. Comparative Expression Profiles of Categorized Surface Antigens

#### 3.2.1. Hematopoietic Stem Cells Markers

Hematopoietic stem cell markers such as CD34, CD133, CD45, and HLADR were studied for their expression. It is evident from the analysis that these markers were found to be sparsely expressed in early and later passages of all sources in all media except for a slight higher expression of CD45 at early passage of omentum fat MSCs cultured in DMEM-F12.

#### 3.2.2. Mesenchymal Stem Cells Markers

The study revealed a similar remarkable expression of these markers CD90, CD105, and CD73 throughout the long term culture condition of all sources in all media, as defined by ISCT [21]. The SSEA4 showed a sparse expression of all sources in all media. However, an increase in expression was identified in DMEM-LG of all sources.

#### 3.2.3. Cell Adhesion Molecules/Surface Enzymes/Side Population

MSCs innate property of transendothelial migration and homing is indebted to the presence of cell adhesion molecules. Current study includes the following cell adhesion molecules: CD29, CD44, CD166, CD106, CD31, CD49d, and CD54, surface enzymes: CD13 and ALDH and side population: ABCG2 and CD117. The sparse expressions of CD106 and CD31 were found in all sources of all media. Similarly, the ALDH expression was found to be lower in all sources of all media except for its moderate expression in both early and later passages of DMEM-F12 for OF. Unlike the expressions of MSC specific markers, the study showed a varying expression in the markers such as CD54, CD49d, and CD117 in early and later passages of all sources in all media, except for its similar expressions of CD54 in early passages of SF and BM, CD 49d in early passages of OF and CD117 in both early and later passages of SF in all media as illustrated (Table 1). On the other hand, the markers such as CD29, CD44, CD166, and CD13 showed remarkable expressions throughout the long term culture condition except for its slight decrease in its expression of CD13 at early passage of OF at Alpha-MEM and DMEM-KO.

## 4. Discussion

Over the past 6 years, there are several reports on variations exhibited in the characterization of cell surface markers at different stages of MSC culture from bone marrow and adipose tissue [3, 10, 13, 20, 22–26], further making cell surface marker expression study an arduous task. The expression profile was identified to change as a function of time in passage and plastic adherence [27, 28]. Hence, there is a lack of thorough understanding of the mechanism underlying stem cell renewal and its functional differentiation. Although, the maintenance of stemness property such as cell proliferation and cell differentiation under long term cultures of different media was studied [9, 19, 29, 30], identification of prospective definitive markers specific to MSCs of the existing contemporary therapeutic adult postnatal sources such as bone marrow and adipose tissue remains elusive. Further, it has not yet been studied what happens to these markers under long term cultures with different media. This formed the basis of our present study.

The impact of different culture media (DMEM-LG, Alpha-MEM, DMEM-F12, and DMEM-KO) exposed to long term culture conditions of MSCs obtained from OF, SF, and BM was analysed in detail for diversified surface antigen until P20 for bone marrow samples and until P25 for adipose tissue samples. Out of the four samples processed from each source, unlike omentum fat and subcutaneous fat, only one sample of bone marrow could grow beyond P20 and rest lost its potential to grow beyond P15. However, on the other hand, the influence of different culture media has not lead to marker expression variations in both early and later passages of all sources except for certain exhibited marker variations seen in CD49d, CD54, and CD117.

These marker variations exhibited in our results in all media was similar to the results of certain previously published [3, 10, 13, 23, 24, 26]. However, further in-depth research is of utmost importance on the cell adhesion molecule that interacts with the cytoskeleton of MSC. This might enhance the understanding of MSC as an instrument of curative therapeutics involved in the applications of neovascularisations, angiogenesis, and treatment of other vascular disorders. This is due to the fact that CD117 serves as an important growth factor that plays a vital role in cell survival, proliferation, and differentiation [31]. CD54 is an endothelial and leukocyte-associated transmembrane protein long known for its importance in stabilizing cell-cell interactions and facilitating leukocyte endothelial transmigration. As a result of these binding characteristics, CD54 has classically been assigned the function of intercellular adhesion [32]. Similarly, CD49d along with the higher expression of its counterpart, CD29, together forming VLA4 is supposed to play a role in mobilization and homing [20, 23, 33, 34].

However, we found a contrary expression pattern in the markers such as CD49d and CD106 when compared to the previously published reports. The literature reports that adipose tissue MSCs expresses CD49d and not CD106, whereas bone marrow MSCs expresses CD106 but not CD 49d. This reciprocal expression pattern is interesting because CD106 is the cognate receptor of CD49d and both these molecules represent part of a receptor-ligand pair that has an important role in hematopoietic stem cell homing to and mobilization from bone marrow [35–37]. However, our study revealed a varying expression pattern of CD49d in all sources including bone marrow and a similar sparse expression of CD106 was obtained in all sources, including adipose tissue. Further research on the discrepancies related to CD49, CD29, and CD106 might throw light as these molecules together play a vital interactive role. Besides, CD13 was also identified to be a potent marker that plays a vital role in angiogenesis and migration [20, 38].

Besides, the similar remarkable expression patterns of cell surface markers such as CD90, CD105, CD73, CD29, CD44, CD166, and CD13 and negative expressions of CD34, CD45, CD133, CD31, and HLADR obtained in our study have been detected with highly consistent patterns of expression on the surface of MSCs by different literatures [3, 22, 24, 39, 40]. This coherence in negative expressions supports the fact that these marker expressions were lost with passage; and subsequent expansion will select for a relatively homogeneous cell population compared with the whole cell population [3, 10, 22, 26, 39–41]. The wealth of knowledge on these markers about their crucial migration and homing evokes that these markers impersonate a CAM that performs these aforesaid functions in MSC. Although the existence and functionality of certain MSC specific markers is known, there is uncertainty among the specificity and functionality of several other markers of MSC, thereby demanding further extensive research.

In addition, this study also analysed for the expression of ALDH and ABCG2 in all sources of tissue and different basal media. This is due to the fact that ALDH isozymes involved in drug resistance and retinoic acid generation would be crucial for the protection of stem cells against toxic endogeneuos and exogeneous aldehydes and for their ability to differentiate [42]; it serves as a key marker for the prediction of therapeutic efficacy of MSCs. Similarly, ABCG2 plays a role in protecting stem cells by increasing their survival capacity and proliferation potential, processes which are fundamental for stem cell maintenance and renewal [43]. In search of a novel marker for prospective isolation of tissue specific MSC, notion of cracking MSCs pluripotency was carried by Gang et al. and coworkers for SSEA-4. In coherence with his reports, our study also showed the expressions of SSEA4. Its expression was identified to be more in omentum fat MSCs and in DMEM-LG medium as compared to other source and media, respectively [44].

## 5. Conclusion

It was demonstrated that the phenotypic characterization of MSCs remained unchanged irrespective of source of tissue, basal media, and extensive culturing. However, further attention on the markers such as CD49d, CD54, CD117, CD29, and CD106 of each source is suggested. Besides, our data clearly shows that any basal media could be used for culturing these sources.

Although this study resolves the enigma that has been circulating all over on the identity of tissue specific cell surface markers, there is a lot more to be explored in all fronts of phenotypic characterization of stem cells for generation of specific MSCs for the specific condition based cell based therapies.

## Figures and Tables

**Table 1:** Comparative analysis of ranges of marker expression profile.

MARKERS	PASSAGE	OF				SF				BM			
		LG	MEM	F12	KO	LG	MEM	F12	KO	LG	MEM	F12	KO
ALDH	EARLY	L	L	M	L	L	L	L	L	L	L	L	L
	LATE	L	L	M	L	L	L	L	L	L	L	L	L
CD29	EARLY	R	R	R	R	R	R	R	R	R	R	R	R
	LATE	R	R	R	R	R	R	R	R	R	R	R	R
CD90	EARLY	R	R	R	R	R	R	R	R	R	R	R	R
	LATE	R	R	R	R	R	R	R	R	R	R	R	R
CD105	EARLY	R	R	R	R	R	R	R	R	R	R	R	R
	LATE	R	R	R	R	R	R	R	R	R	R	R	R
SSEA4	EARLY	S	S	S	S	L	S	S	S	L	S	S	S
	LATE	L	S	S	S	L	S	S	S	L	L	S	S
CD34	EARLY	S	S	L	S	S	S	S	S	S	S	S	S
	LATE	S	S	S	S	S	S	S	S	S	S	S	S
CD45	EARLY	S	S	L	S	S	S	S	S	S	S	S	S
	LATE	S	S	S	S	S	S	S	S	S	S	S	S
CD133	EARLY	S	S	S	S	S	S	S	S	S	S	S	S
	LATE	S	S	S	S	S	S	S	S	S	S	S	S
CD31	EARLY	S	S	S	S	S	S	S	S	S	S	S	S
	LATE	S	S	S	S	S	S	S	S	S	S	S	S
HLA-DR	EARLY	S	S	S	S	S	S	S	S	S	S	S	S
	LATE	S	S	S	S	S	S	S	S	S	S	S	S
CD44	EARLY	R	R	R	R	R	R	R	R	R	R	R	R
	LATE	R	R	R	R	R	R	R	R	R	R	R	R
CD73	EARLY	R	R	R	R	R	R	R	R	R	R	R	R
	LATE	R	R	R	R	R	R	R	R	R	R	R	R
CD13	EARLY	R	H	R	H	R	R	R	R	R	R	R	R
	LATE	R	R	R	R	R	R	R	R	R	R	R	R
CD54	EARLY	R	R	H	R	M	M	M	M	M	M	M	M
	LATE	H	H	H	M	R	H	H	M	M	M	M	L
CD49d	EARLY	M	M	M	M	H	M	M	M	H	L	L	L
	LATE	R	M	H	M	H	H	H	H	S	M	L	L
CD117	EARLY	M	H	H	M	H	H	H	H	M	H	H	H
	LATE	H	H	H	H	H	H	H	H	H	H	H	M
ABCG2	EARLY	S	S	S	S	S	S	S	S	S	S	S	L
	LATE	S	S	S	S	S	S	S	S	S	S	S	L
CD166	EARLY	R	R	R	R	R	R	R	R	R	R	R	R
	LATE	R	R	R	R	R	R	R	R	R	R	R	R
CD106	EARLY	L	S	S	S	S	S	S	S	S	S	S	S
	LATE	S	S	S	S	S	S	S	S	S	S	S	S

Expression ranges: R: remarkable (>90%); H: high (75–89%); M: moderate (40–74%); L: low (11–39%); S: sparse (<10%). Early (P1, P3, and P5), late (P9, P12, P15, and P20); OF: omentum fat; SF: subcutaneous fat; BM: bone marrow; LG: low glucose; MEM: minimum essential media; KO: knock-out.

## Abbreviations

Alpha-MEM: Alpha minimal essentialmedia
BMI: Body mass index
CD: Cluster of differentiation
DEAB: Diethylaminobenzaldehyde
DMEM: Dulbecco's modified eagle's medium
DMSO: Dimethyl sulfoxide
FBS: Fetal bovine serum
KO: Knock out
LG: Low glucose
MSC: Mesenchymal stem cells
PBS: Phosphate buffered saline.
